# Research advances in drug therapy of endometriosis

**DOI:** 10.3389/fphar.2023.1199010

**Published:** 2023-06-21

**Authors:** Jianyou Shi, Xin Tan, Guimei Feng, Yuan Zhuo, Zhongliang Jiang, Srikanth Banda, Lin Wang, Wei Zheng, Lu Chen, Dongke Yu, Chun Guo

**Affiliations:** ^1^ Department of Pharmacy, Sichuan Academy of Medical Sciences and Sichuan Provincial People’s Hospital, School of Medicine, University of Electronic Science and Technology of China, Chengdu, China; ^2^ Personalized Drug Therapy Key Laboratory of Sichuan Province, School of Medicine, University of Electronic Science and Technology of China, Chengdu, China; ^3^ Center for Reproductive Medicine, Department of Obstetrics and Gynecology, Sichuan Provincial People’s Hospital, University of Electronic Science and Technology of China, Chengdu, China; ^4^ Chinese Academy of Sciences Sichuan Translational Medicine Research Hospital, Chengdu, China; ^5^ Pharmacy College, Chengdu University of Traditional Chinese Medicine, Chengdu, China; ^6^ Miller School of Medicine, University of Miami, Miami, FL, United States; ^7^ Department of Chemistry and Biochemisty, Florida International University, Miami, FL, United States; ^8^ College of Food and Bioengineering, Xihua University, Chengdu, China

**Keywords:** endometriosis, pathogenesis, medical treatment, gonadotropin-releasing antagonist, aromatase inhibitor

## Abstract

Endometriosis is one of the most common benign gynecological disorders in reproductive-aged women. The major symptoms are chronic pelvic pain and infertility. Despite its profound impact on women’s health and quality of life, its pathogenesis has not been fully elucidated, it cannot be cured and the long-term use of drugs yields severe side effects and hinders fertility. This review aims to present the advances in pathogenesis and the newly reported lead compounds and drugs managing endometriosis. This paper investigated Genetic changes, estrogen-dependent inflammation induction, progesterone resistance, imbalance in proliferation and apoptosis, angiogenesis, lymphangiogenesis and neurogenesis, and tissue remodeling in its pathogenesis; and explored the pharmacological mechanisms, constitutive relationships, and application prospects of each compound in the text. To date, Resveratrol, Bay1316957, and bardoxifene were effective against lesions and pain in controlled animal studies. In clinical trials, Quinagolide showed no statistical difference with the placebo group; the results of phase II clinical trial of the IL-33 antibody have not been announced yet; clinical trial stage III of vilaprisan was suspended due to drug toxicity. Elagolix was approved for the treatment of endometriosis-related pain, but clinical studies of Elagolix for the pretreatment of patients with endometriosis to before In vitro fertilization treatment have not been fulfilled. The results of a clinical study of Linzagolix in patients with moderate to severe endometriosis-related pain have not been disclosed yet. Letrozole improved the fertility of patients with mild endometriosis. For endometriosis patients with infertility, oral GnRH antagonists and aromatase inhibitors are promising drugs, especially Elagolix and Letrozole.

## 1 Introduction

The presence of functional endometrial glands and stroma growing outside of the uterine cavity is termed as Endometriosis. The ectopic endometrium could invade any part of the body, but the pelvic organs, the wall peritoneum, the ovaries, and the uterosacral ligament are the most invaded. The World Endometriosis Society (WES) proposed that endometriosis is a disease with multiple pathological factors including specific genetic features, immune abnormality, hormone imbalance, hemorrhagic factors, and organ-dependent disease. Endometriosis is one of the most common benign gynecological disorders among premenopausal women, and the relevant major symptoms gradually increase the rates of secondary dysmenorrhea and infertility. Studies showed 10%–15% of childbearing-age women have been affected by pelvic endometriosis and 30%–50% of the patients are affected by the symptoms of chronic pelvic pain and/or infertility (Fukunaga, 2001; Burney and Giudice, 2012; Dyson et al., 2014). The incidence of endometriosis has increased in recent years and the treatment for the disease costs $70 billion per year in the United States alone (Simoens et al., 2012).

Current endometriosis treatment options include medication, surgery, surgery combined with medication, and assisted reproductive technology. Giving medication to alleviate the relevant symptoms is preferred for patients without surgery indication. The First-line drugs include non-steroidal anti-inflammatory drugs (NSAIDs), progestins, and oral contraceptives (OCs). The Second-line drugs include gonadotropin-releasing antagonists (GnRH-a) and levonorgestrel intrauterine release system (LNG-IUS). However, application of the a mentioned medicines such as GnRH-a exhibits severe side effects including estrogen deficiency symptoms and ovation inhibition. Therefore it is not suitable for long-term use, especially for patients with fertility needs. What’s more, there is no cure for endometriosis. Due to these limitations (Dunselman et al., 2014), there is an urgent need for developing new therapeutic agents that can effectively alleviate the endometriosis symptoms without impeding the patient’s fertility. In this review, we summarized the lead compounds for endometriosis treatment and the promising drug clinical trials in recent years.

## 2 The pathogenesis of endometriosis

### 2.1 The generation of endometriosis tissue

**FIGURE 1:**
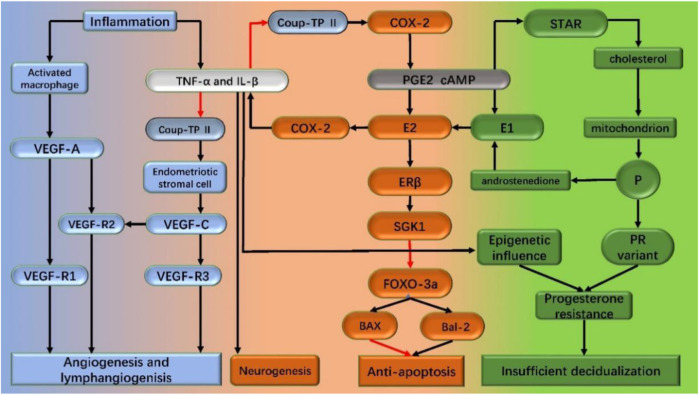
Mechanism of endometriosis pathogenesis in the text. Inflammation is the central link in the pathogenesis of endometriosis. Inflammation promotes the synthesis of estrogen. Increased estrogen level promotes apoptosis and the secretion of inflammatory factors like TNF-a and IL-1β, thus forming a vicious circle. Under the stimulation of the inflammatory factors on epigenetic change and/or progesterone receptor mutations, progesterone resistance occurs, which leads to decidualization insufficiency. Driven by inflammation, angiogenesis and lymphangiogenesis increased, neurogenesis, and endometriosis lesions formed and continued to develop. Black arrows: stimulation; red arrows: inhibition; TNF-a: tumor necrosis factorα; IL-1β: interleukin-1β; COX-2: cyclooxygenase type 2; StAR: steroid acute response protein (StAR); ER β: estrogen receptor β; SGK1: serum and glucocorticoid-regulated kinase FOXO-3a:forkhead box class o 3a; PGE2: prostaglandin E2; cAMP: cyclic adenosine monophosphate; P: progesterone; PR: progesterone receptor; VEGF: vascular endothelial growth factor.

Different theories have emerged to explain the pathological mechanism of endometriosis. The most widely accepted hypothesis is the menstrual retrograde theory. According to this hypothesis, the menstrual blood transports viable endometrial fragments through the fallopian tube to the peritoneal cavity, where those fragments can be implanted, developed, and in certain specific cases, invade other pelvic tissues during menstruation. In 1927, Sampson et al. (Sampson, 1927) first proposed the menstrual retrograde theory and a large number of clinical evidence supported the theory. Halme et al. (Halme et al., 1984) not only confirmed the menstrual retrograde theory but further demonstrated that blood was visible in the peritoneal fluid under laparoscopy in 90% of women during their menstrual periods. While among the patients diagnosed with tubal obstruction, only 15% of them showed traces of blood in the peritoneal cavity. Chapron et al. (Chapron et al., 2006) explored the anatomical distribution of deep infiltration lesions in 426 endometriosis patients who suffered pelvic pain from 1992 to 2004, the results showed that the distribution of deep-invasive endometriosis lesions was asymmetric. The authors believe that the anatomical differences between the left and right sides of the pelvis and the flow of peritoneal fluid contributed to the asymmetric lesion pattern, which confirmed Sampson’s theory of menstrual reflux. The asymmetry in the distribution of endometriotic lesions is related to the anatomy of the abdominal and pelvic cavit and the mode of peritoneal fluid flow (Figure 1).

On the flip side, Redwine et al. (Redwine, 2002) found that the endometriosis lesions were different from autologous transplantation, the auto-transplanted tissues are identical to *in situ* tissues and remain structurally and functionally normal for decades, while the endometriosis lesions are profoundly different from normal endometrium. Their differences include the clonal origin, endometrial enzyme activities, protein expression levels, morphological, and histological features. There are two scenarios regarding the difference between endometriosis and auto-transplanted tissues.

In conclusion, these theories can generally be divided into two categories: one of which proposes that the implants come from endometrium and the rest believe the implants come from other tissues.

### 2.2 Genomic and epigenetic changes

#### 2.2.1 Genomic changes

Clinical observation revealed that endometriosis may be a multi-gene manipulated phenotype that tends to be inherited. There was a study reported that mothers and sisters of women with severe endometriosis symptoms were seven times more likely to develop endometriosis than their partner’s leading female relatives (Simpson et al., 1980). Consistent with this observation, familial endometriosis cases tend to be more severe and have an earlier onset of symptoms compared to the disseminated cases (Kennedy et al., 1995). According to the study of 3,096 pairs of twins, the genetic rate of endometriosis, or the proportion of disease variation caused by genetic factors, was estimated at about 52%. These familial studies point to a genetic predisposition to the disease (Treloar et al., 1999).

Sapkota et al. identified five novel loci (FN1, CCDC170, ESR1, SYNE1 and FSHB) significantly associated with endometriosis risk through a meta-analysis concluding 11 genome-wide association case-control data sets, which included 17,045 endometriosis cases and 191,596 controls. And conditional analysis resulted in 19 independent single nucleotide polymorphisms (SNPs) significantly associated with endometriosis, which together explain 5.19% of variance in endometriosis. The results imply gene variants may play important roles in sex steroid hormone signaling and endometriosis pathogenesis (Sapkota et al., 2017).

#### 2.2.2 Epigenetic changes

##### 2.2.2.1 Covalent Modification

Studies of endometriosis mesenchymal cells provided clues to epigenomic alterations leading to heritable traits without altering DNA sequences (Peiris et al., 2018; Koninckx et al., 2019). The abnormal hypomethylation pattern in the promoter region of estrogen receptor beta (ERβ) seems to have a critical effect in endometriosis associated epigenetic abnormalities (Bulun et al., 2019; Yilmaz and Bulun, 2019). The estrogen receptor changing from α (ERα) to β (ERβ) altered the estradiol exposure response which include downregulate the induction of progesterone receptor (PR) in stromal cells and further resulted in the PR subtype expression decrease. The decrease of the ERα/ERβ ratio may induce the chromatin conformation change in the endometrial mesenchymal stem cells (eMSC) which triggers the relevant genes’ expression change and finally lead to the development of acquired genetic progesterone resistance. The endometriosis lesions are formed by the deposition of epigenetically defective eMSCs that are repeatedly exposed to the peritoneum during repeated menstrual periods. Post lesion formation, the estrogen exposure of ERβ and other pro-inflammatory factors increases inflammation and promotes the survival of endometriotic cells within the lesion (Yilmaz and Bulun, 2019). The endometriosis mesenchymal cells express steroidogenic enzymes and sulfate esterase enzymes resulting in the accumulation of a significant amount of estradiol locally (Rižner, 2016). Inflammation and increased prostaglandin 2 (PGE2) induce the expression of steroid enzymes, aromatase, and steroid acute response protein (StAR) through the promoter region of steroid synthesis factor 1 (NR5A1) (competing with chicken ovalbumin upstream promoter transcription factor II (NR2F2)). Activation of this cascade reaction further exacerbates inflammation through increasing local estradiol communication with ERβ and creates a vicious cycle by increasing cyclooxygenase 2 (COX-2) levels.

##### 2.2.2.2 Genome-Wide Methylation Studies

The advancement of next-generation DNA sequencing technologies in recent years makes it possible to investigate the genome-wide methylation pattern. Houshadarn et al. (Houshdaran et al., 2016) analyzed the genome-wide methylation pattern in women with endometriosis at three different periods. The authors reported an overall predominance of hypomethylation (61%) and a small fraction of hypermethylated loci (10%) in the biopsies obtained from endometriosis patients. Abnormal methylation patterns were found to interfere with a variety of physiological activities including the regulation of the cell cycle, immune response and the subsequent inflammation reactions, steroid hormone response, cell migration, and gene expressions. The aforementioned discoveries were consistent with other genome-wide methylation and transcriptome studies (Afshar et al., 2013).

### 2.3 Altered hormonal regulatory system

#### 2.3.1 Estrogen dependent inflammation induction

The molecular and cellular markers of inflammation include cytokines, prostaglandins production, and immune cell infiltration (Lebovic et al., 2001). Also, the endometrial stromal cells are one of the sources of cytokines and prostaglandins (Tseng et al., 1996; Ota et al., 2001). The repeated bleeding and migration of macrophages to the lesion site for blood pigments removal also contribute to inflammation and adhesion. Studies showed that the use of GnRH mimics and/or aromatase inhibitors prevented estradiol from entering endometriosis tissue, ther alleviating and even stopping symptoms like pain among the patients (Monsivais et al., 2014). Based upon the aforementioned evidence, the endometriosis inflammatory process is thought to be induced by estradiol and mediated by estrogen receptor β (ERβ) (Takayama et al., 1998; Monsivais et al., 2014; Chandler et al., 2015; Monsivais et al., 2016), and correlates with the estrogen receptor (ER) expression levels (Bukulmez et al., 2008).

Because of the strong estrogen-mediated effects on the disease and the high expression levels of ERβ in affected tissues, it is critical to determine ERβ transcriptional targets in endometriosis. Previous studies have discovered the expression of ERβ-driven transcription genes in the stromal cells of endometriosis (Monsivais et al., 2014). One of the identified genes was serum and glucocorticoid-regulated kinase (SGK1) which is overexpressed in the endometriosis tissue and contains an ERβ binding site in its promoter region. Related studies have shown that SKG1 not only has anti-apoptotic effects, but also can regulate the expression of the pro-apoptotic factor FOXO3a (Brunet et al., 2001; Zhang et al., 2001; Leong et al., 2003) Diana Monsivais et al. (Monsivais et al., 2016) verified SGK1 is a transcriptional target of ERβ upregulation in endometrial tissue which promotes the survival of endometriosis stromal cells.

Aromatase is the rate-limiting enzyme that converts androgens to estrogens which are not expressed in normal endometrium but highly expressed in both ectopic and *in situ* endometrium in patients with endometriosis. Therefore, it is an important cause of increased estrogen synthesis in the endometriosis ectopic lesions (Ferrero et al., 2014). Aromatic enzymes convert the ovary and adrenal gland sourced androstenedione into estrone (E1), and 17β-hydroxysteroid dehydrogenase (17β-HSD) further converts E1 into estrogen (E2). The cyclic adenosine monophosphate (cAMP) analogs or prostaglandin E2 (PGE2) upregulates the aromatase expression in ectopic endometrial stromal cells, and PGE2 expression is regulated via cyclooxygenase type 2 (COX-2). In addition, PGE2 upregulates the expression of StAR which promotes the entry of cholesterol into mitochondria for the synthesis of sex hormones (Mori et al., 2018).

Inflammation is the central process of endometriosis which causes various symptoms including pain, adjacent tissue remodeling, fibrosis induction, adhesion formation, and infertility. The aforementioned studies demonstrated that the inflammatory reaction environment facilitates the E2 synthesis in ectopic lesions, while the local inflammatory reaction is correlated to high level estrogen exposure, to create a vicious circle.

#### 2.3.2 Progesterone resistance

A critical feature of endometriosis is insufficient decidualization (Burney et al., 2007; Aghajanova et al., 2011; Dyson et al., 2014) which has been confirmed associated with progesterone resistance (Zeitoun et al., 1998). Although the molecular evidence of progesterone resistance was first observed in endometriosis epithelial cells, the stromal decidual defects mechanism was found to be dominant (Zeitoun et al., 1998; Kim et al., 2013). Besides, the epigenetically programmed faulty mesenchymal stem cells in endometriosis also result in differentiation defects (Barragan et al., 2016; Gargett et al., 2016).

The key feature of the low response of endometriosis to progesterone is the insufficiency of progesterone receptor B transcription, translation and biological activity. This defect may be congenital due to the progesterone receptor gene variant (DʼAmora et al., 2009), or acquired through the epigenetic factors of an inflammatory milieu (Patel et al., 2017).

### 2.4 Proliferation and apoptosis imbalance

#### 2.4.1 Proliferation

The epithelial cells in the eutopic endometrium proliferate in response to estrogen exposure (Kim et al., 2013). Although the high proliferation rate may contribute to the accumulation of mutations in the epithelial cells in endometrial tissues (Suda et al., 2018), most endometriosis implants lack epithelial cells but are mainly composed of mesenchymal cells. In contrast to the epithelial cells, the mesenchymal cells are not inclined to proliferate extensively and are not prone to accumulate mutations. Therefore, the endometrium or endometriosis mesenchymal stromal cells have limited proliferative activity. As estrogen receptor was concerned, ERβ was recognized to be more abundant in ectopic endometriotic lesions than ERα, and high expression of ERβ could lead to the production of CCL2 via NF-κB signaling, which resulted in macrophages being recruited to the ectopic milieu. Recruited macrophages were also proved to promote the proliferation and clonogenic ability of endometrial stromal cells in an *in vitro* co-culture assay (Gou et al., 2019).

But the proliferative activity of endometriosis tissue was reported significantly reduced compared to the normal endometrial tissue, with the exception of endometriosis tissues on the abdominal wall (Béliard et al., 2004). One hypothesis to explain this exception is that the aromatase activity of adipose fibroblasts in subcutaneous adipose tissue may be pro-increases the transplanted endometriosis tissues proliferation rate (Bulun and Simpson, 1994). Notably, the aromatase activity in human subcutaneous adipose tissue is significantly higher than that in omental or visceral (e.g., subperitoneal) adipose tissue (Bulun et al., 2005). As we know, aromatase could promote the synthesis of estrogen.

#### 2.4.2 Reducing apoptosis

Previous studies showed that the apoptotic endometrial stroma and epithelial cell proportions in endometriosis patients were significantly lower compared to their normal endometrium counterparts (Dmowski et al., 2001; Béliard et al., 2004). Such a phenomenon may be associated with the abnormal local estradiol level. The Monsivais group demonstrated that the estrogen beta receptor (ERβ) mediates the anti-apoptotic effect of estradiol in endometriosis mesenchymal cells (Monsivais et al., 2016). Although apoptosis is regulated by several genes during the menstrual cycle (Bax, c-myc, and P53 induce apoptosis, whereas sentrin, B-cell lymphoma/leukemia-xl, and Bcl-2 inhibit apoptosis), changes in endometrial apoptosis during the menstrual cycle appear to be mainly regulated by ovarian steroids that upregulate and downregulate the expression of Bcl-2 and Bax, and the expression level of Bax depends on the role of Bcl-2 (Lagana et al., 2019). The anti-apoptotic gene Bcl-2 shows upregulation in both ectopic and *in situ* endometrium of affected women (Jones et al., 1998), thus, reducing apoptosis of endometrial mesenchymal and epithelial cells.

### 2.5 Angiogenesis, lymphangiogenesis and neurogenesis

The formation of blood vessels and their steroid hormone-dependent correlation are commonly observed in endometriosis lesions cases. Study shows various cytokines such as IL-17A may enhance angiogenesis and thus facilitate the plant growth of endometriotic lesions (Ahn et al., 2015). The vascular endothelial growth factor (VEGF) family is involved in the development of ectopic endometriosis (Numao et al., 2011; Djokovic and Calhaz-Jorge, 2014). Some studies also observed the occurrence of lymphangiogenesis in endometriosis lesions, which means new lymph vessels were generated from the original blood vessels (Keichel et al., 2011; Reichelt et al., 2012; Jerman and Hey-Cunningham, 2015), and lymphangiogenesis growth factors including VEGF-C and VEGF-D increased significantly from the peritoneal lesions. (Takehara et al., 2004; Keichel et al., 2011).

VEGF-A plays a central role in both physiological and pathological angiogenesis and may be involved in both the etiology and maintenance of peritoneal endometriosis. Previous studies showed VEGF-A binds to two different receptors including VEGF receptor 1 (VEGFR1) and VEGFR2. The VEGFR1 is also known as fms-like tyrosine kinase 1 (flt1), which is expressed in monocytes, macrophages, and vascular endothelial cells. Also, macrophages are responsible for lymphangiogenesis and other studies reported that VEGFR1 signaling in macrophages promotes wound healing associated with lymphangiogenesis (Okizaki et al., 2016). Based on the aforementioned findings, the authors hypothesized that VEGFR1 signaling is involved in lymphangiogenesis and the development of endometriosis. The VEGF receptor 2 (VEGFR2) also known as kinase insert domain-containing receptor (KDR), it is expressed by endothelial cells (Shibuya, 2008). Mice lacking VEGFR2 (Kdr^−/−^) could not develop blood vessels and are dead in uterus, suggesting that VEGFR2 plays a dominant role in the development of vascular system (Shalaby et al., 1995).

Vascular endothelial growth factor-C (VEGF-C) is a well-known growth factor responsible for angiogenesis, especially in lymphangiogenesis too. Studies showed (Bulun et al., 2019) the pro-inflammatory cytokines (such as IL-1β and TNF-α) inhibit the expression of COUP-TFII in endometrial stromal cells, resulting in the secretion of vascular endothelial growth factor-C (VEGF-C). The COUP-TFII is a multifunctional transcription inhibitor, which has been confirmed to inhibit the expression of aromatase, cyclooxygenase-2 (COX-2), and angiopoietin in the endometriosis stromal cells. The binding of VEGF-C to vascular endothelial growth factor receptor 2/receptor 3 (VEGFR2/R3) on lymphatic vessel endothelial cells induces lymphangiogenesis which further leads to the formation of endometriosis lesions. Therefore, it can be concluded that inflammation promotes angiogenesis and lymphangiogenesis in endometriosis. The formation of lymphatic vessels provides a new approach for the immune cells to infiltrate into endometriosis lesions.

Neurogenesis provides a mechanism that could explain the association between endometriosis and pain pathways. IL-1β as an inflammatory factor could stimulate nerve growth factor expression in endometriosis directly, thus it is associated with local neurogenesis adjacent to endometriosis lesions and so severe deep dyspareunia (Peng et al., 2020).

### 2.6 Organizational restructuring

The implanted tissues’ viability is critical for the initial attachment of endometrial tissue fragments to the pelvic peritoneum and the establishment of subperitoneal endometrial lesions (Witz et al., 2001). Once the initial implantation of endometrial tissue fragments occurs, the limited cell proliferation and tissue growth may be necessary for the long-term survival of the implanted tissue. The estrogen exposure induces inflammation and results in the significant remodeling of peritoneal and subperitoneal tissues, like the adipose tissue. Studies showed the high metalloproteinase activity of endometriosis implants plays a critical role in inducing the surrounding tissues’ remodeling (Bruner et al., 1997). Indeed, the fibrosis of the surrounding tissues is a hallmark of peritoneal or ovarian endometriosis implant formation (Bulun et al., 2019). In sum, Estrogen-driven inflammation appears to be the central process shaping the endometriosis pathology.

## 3 Classification of the drugs for endometriosis treatment

The goal of drug therapy for endometriosis is to relieve pain symptoms and improve fertility. Studies showed that long-term drug management post-surgical operation delays the recurrence of endometriosis symptoms among patients. At present, drugs commonly used to treat endometriosis include NSAIDS, progestogen, combined oral contraceptives, GnRH-a, GnRH-antagonist (GnRH-A), Selective Progesterone Receptor Modulators (SPRM), Selective Estrogen Receptor Modulators (SERM), an aromatase inhibitor (AI), etc. And there are some new products such as prostaglandin E2 (PGE2) receptor antagonists, resveratrol, IL-1 antagonist, and Dopamine receptor antagonist. The most promising drug is GnRH-A (Table 1).

**TABLE 1:** Clinical trials of drugs for endometriosis.

Medicine	Target/mechanism (mode of delivery)	Therapy	Phase		ClinicalTrials.gov
Linzagolix	Gonadotropin-releasing hormone antagonist	Endometriosis	Ⅲ	June 2019 to July 2022	NCT03992846
Vilaprisan	Selective progesterone receptor modulator	Myoma of uterus	Ⅲ	January 2018 to November 2021	NCT03400943
Quinagolide	Dopamine receptoragonists	Endometriosis pain	Ⅱ	January 2018 to August 2023	NCT03692403
IL-1	Immune adjustment	Endometriosis pain	Ⅰ	June 2020 to January 2022	NCT03991520

### 3.1 Progestogen

Progesterone drugs exhibit therapeutical effects on endometriosis through multiple mechanisms including modulating estrogen receptors, inhibiting inflammatory responses, preventing endometrial proliferation and promoting the apoptosis of endometriosis cells. Progesterone also reduces oxidative stress by reducing or eliminating uterine bleeding in endometriosis lesions (Vercellini et al., 2011). In addition, these drugs also stimulate the atrophy or regression of endometrial lesions, inhibit angiogenesis, and reduce the expression of matrix metalloproteinases, thereby reducing the invasive ability of endometriosis implants (Gezer and Oral, 2015; Casper, 2017). Finally, they reduce the frequency and amount of pulsatile gonadotropin-releasing hormone (GnRH) release; this leads to a decrease in follicle stimulating hormone (FSH) and luteinizing hormone secretion (Gezer and Oral, 2015), thereby establishing a low-estrogen environment that inhibits endometriosis and prevents progression of the disease (Casper, 2017). At present, synthetic progesterone is mainly divided into 19-desmethyltestosterone derivatives, progesterone derivatives, and spironolactone according to the source.

#### 3.1.1 Dinogestrel

**FIGURE 2:**
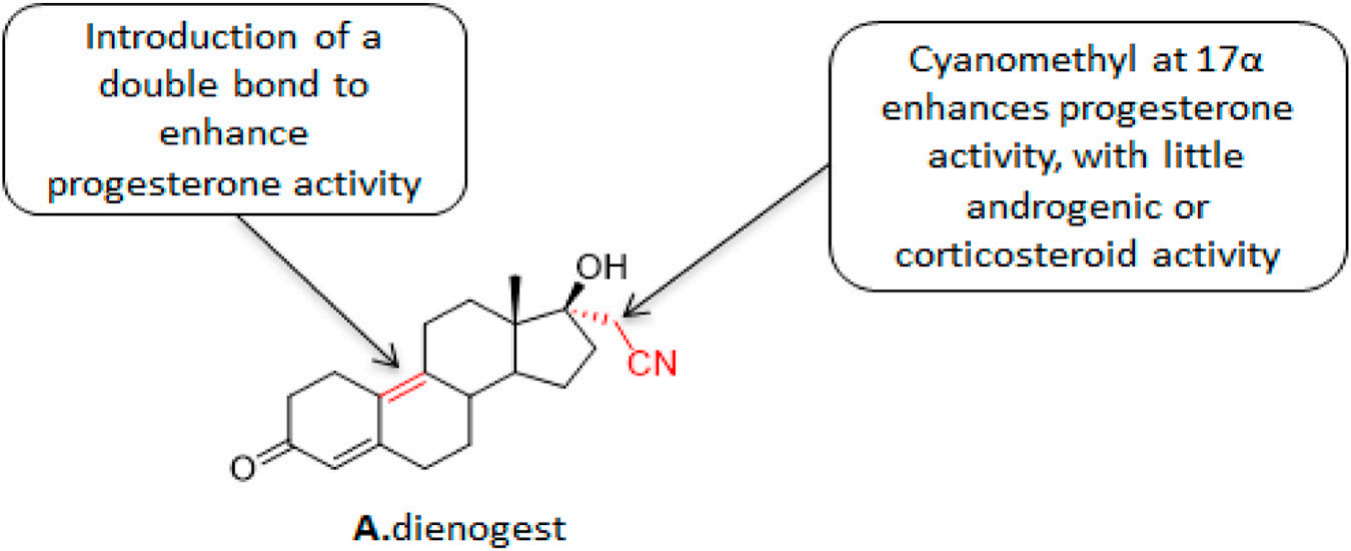
Structure of dienogestrel.

Dinogestrel (DNG) (Figure 2) is a newly marketed fourth-generation selective progesterone that combines the pharmacological properties of 19-desmethyltestosterone and progesterone derivatives. The characteristics of 19-desmethyltestosterone derivatives make DNG highly selective to endometrial progesterone receptors and exhibit an efficient progesterone-like effect. Its pharmacokinetics has the advantages of a short half-life, high oral bioavailability, and no accumulation under *in vivo* conditions. The characteristics of progesterone derivatives make DNG exhibits good tolerance in patients, anti-androgen effect, moderate inhibition of hypothalamus-pituitary-ovarian axis, moderate reduction of estrogen level, and with limited effects on the metabolisms of estrogen, glucose, salt, and lipid in patients. The structure-activity relationship studies on DNG showed that the introduction of double bonds at position 9 enhanced the progestogen-like activity of the lead compound. Replacing the alkyne with a cyanomethyl at position 17α enhanced the progesterone-like activity with almost zero androgen- or corticosteroids-like activities. The *in vitro* studies showed that DNG exhibited moderate affinity to progesterone receptors which was about 10% of the natural progesterone affinity. DNG also exhibits a strong anti-androgen activity with zero glucocorticoids- or halocorticosteroids-like effects. What’s more, DNG does not activate either estrogen receptor α or β. (Foster and Wilde, 1998). Under *in vivo* conditions, DNG exhibits critical pregnancy-like effects. It inhibits the release of gonadotropin, but does not exhibit glucocorticoids-, mineral glucocorticoids-, or significant estrogen-like effects (Foster and Wilde, 1998).

The clinical studies further demonstrated that DNG exhibited better therapeutic effects in endometriosis patients compared to placebo, which was comparable to GnRH agonists and with a better tolerance rate (Kohler et al., 2010; Strowitzki et al., 2010). The progesterone-related side effects are still likely to occur during the DNG treatment with the an incidence rate around 15%. Most of the side effects occur in the first few months of treatment, mainly include abnormal uterine bleeding, headache, constipation, nausea, tidal fever, and weight gain. Also, breast discomfort, personality changes, and libido loss have also been reported (Momoeda et al., 2009). In conclusion, DNG is the only oral progesterone drug specifically designed for the endometriosis treatment. DNG is effective in alleviating the pain symptoms associated with endometriosis such as dysmenorrhea, premenstrual pelvic pain, difficulty with intercourse, and chronic pelvic pain. It is mainly given to patients with mild endometriosis symptoms but shows poor therapeutic effects in patients with severe symptoms. However, the DNG dosing cycle is long, usually 6 months of continuous medication, and it is not suitable for infertile patients due to ovulation inhibition. Therefore, there are space for the further optimization of DNG for the endometriosis treatment.

### 3.2 Gonadotropin-releasing hormone agonist (GnRH-a)

GnRH is a decapeptide compound secreted by the hypothalamus which regulates the reproductive function of the body. It is released into the pituitary portal blood system in a pulsatile manner and acts on the gonadotropin-releasing hormone receptor (GnRH-R). The GnRH-a is a type of drug that has a high affinity to GnRH receptors with a long half-life under *in vivo* conditions. At the initial stage post GnRH-a given, the FSH and LH release were promoted which further increased sex hormone synthesis. After 5–10 days of continuous dosing, GnRH-a binds to most of the GnRH-R in the pituitary therefore blocking the binding of endogenous GnRH to GnRH-R, which resulted in the pituitary gonadotropin secretion decrease, thereby reducing the level of steroid hormones and inducing low estrogen status. The most common adverse reaction of GnRH-a is osteoporosis. ‘It is recommended that clinicians start GnRH-a treatment with a prescription for hormone supplementation to prevent bone loss and hypoestrogenic symptoms during treatment ' and that this does not reduce the effect of pain treatment (Dunselman et al., 2014). The commonly used gonadotropin-releasing hormone agonists are: gonarelin, gosserelin acetate, etc.

### 3.3 Gonadotropin-releasing antagonist (GnRH-A)

GnRH-A competitively binds to the GnRH-R on the pituitary, blocks the endogenous GnRH binds to the GnRH-R, and further reduces the secretion of endogenous gonadotropin in a few hours after medication without ‘flare up’ effect. Besides, GnRH-A has a short dosing period, so it does not cause a decrease in pituitary reactivity after discontinuing its use. The pituitary-gonad axis system usually needs only 2–4 days to restore its function after stop taking GnRH-A. However, taking GnRH-A is highly dose-dependent (Klingmüller et al., 1993). The commonly used GnRH-A is divided into two categories, one is peptide gonadotropin releasing antagonists, and the other is non-peptide GnRH-A. Studies have shown that gonadotropin-releasing antagonists may be an important research direction for the future treatment of endometriosis (Donnez and Dolmans, 2021).

#### 3.3.1 Elagolix

**FIGURE 3:**
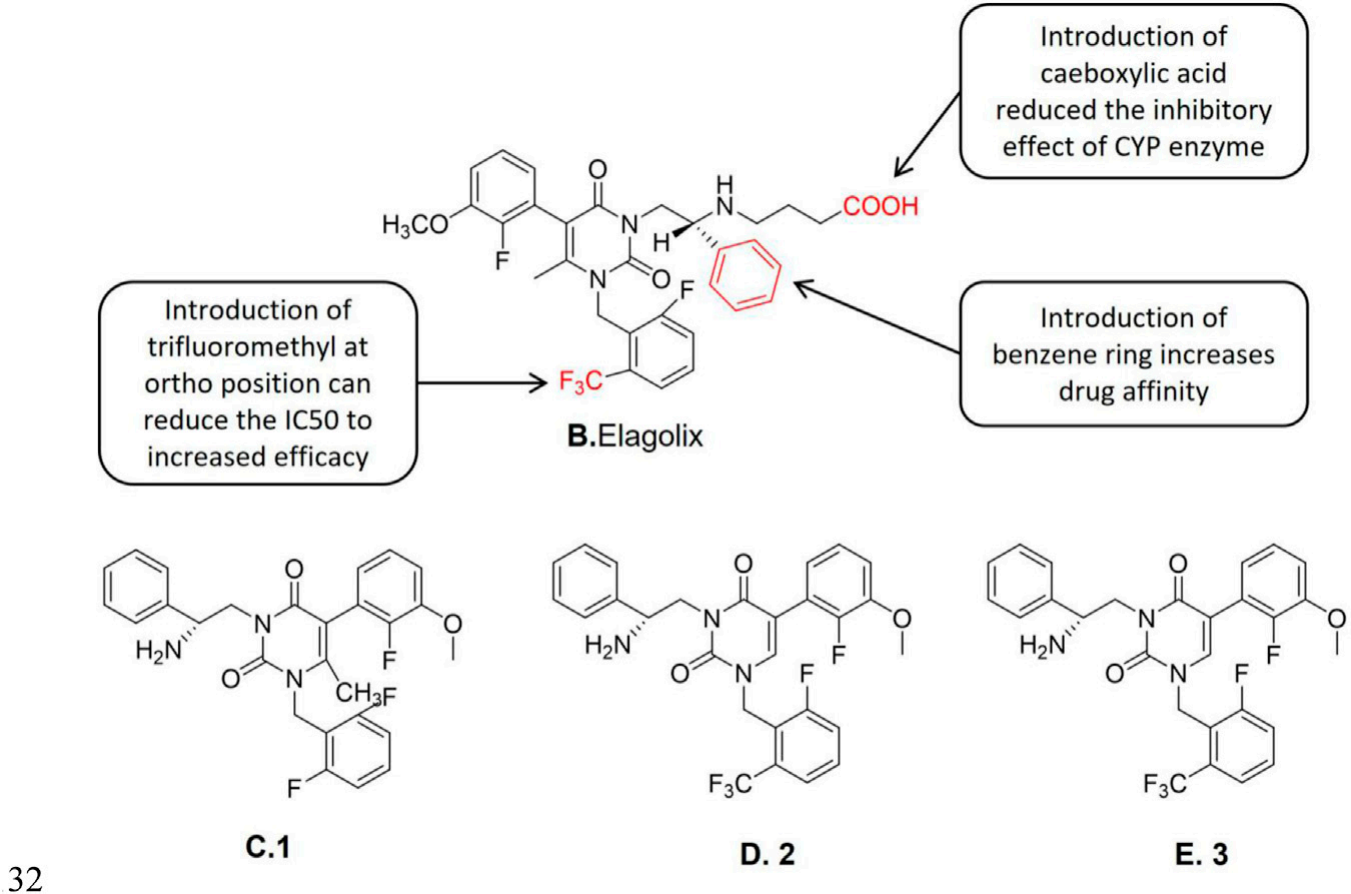
Structure-activity relationship of Elagolix and 1, 2 and 3 structure.

Elagolix (Figure 3B) is a non-peptide gonadotropin releasing antagonist, it is an uracil-phenylethylamine compound containing butyric acid, which is a new oral GnRH-A developed in recent years. Tucci et al. (Tucci et al., 2005) found that 3-[(2R)-amino-2-phenylethyl]1-(2,6-difluorophenyl)-5-(2-fluoro-3-methoxyphenyl)-6-methylpyrimidine-2,4-dione,1 (NBI-42902, Figure 3C) is a weak oral effective human gonadotropin-releasing hormone receptor (hGnRH-R) antagonist. The structure-activity relationship studies showed that when one of the two fluorine groups was replaced by a larger substituent (such as the trifluoromethyl group), its affinity with GnRH-R was improved and the duration of the drug action was also significantly prolonged. The efficacy duration of Elagolix was longer than that of NBI-42902. The structures of the new compounds after the aforementioned structure optimization are represented in Figure 3 as 2 (Figure 3D) and 3 (Figure 3E), respectively. The external racemes of 2 and 3 were tested separately to clarify the role of C-2 stereocenters. Studies showed that the new compound is the unique physiologically related enantiomers (2: IC_50_ (hGnRH-R) = 21 nM, 3: IC_50_ (hGnRH-R) = 2.43 μM, Boselin analysis), and its IC_50_ value shows that 2 is more active *in vivo* than 3 (Panknin et al., 2020). A follow-up study by Sasaki et al. (Sasaki et al., 2003) reported that 3 was an effective human gonadotropin-releasing hormone receptor antagonist. Further studies demonstrated 3 was a potent inhibitor of the CYP3A4 enzyme. Related studies showed that the introduction of butyric acid significantly reduced the inhibition of CYP enzymes compared to the introduction of other carboxyl-containing groups (Chen et al., 2008a) in the aforementioned two derivatives. However, the butyric acid derivatives still preserve the GnRH-R potency. Chen et al. (Chen et al., 2008b) found that the trifluoromethyl structure in Elagolix enhances its hGnRH-R antagonistic effect. The introduction of butyric acid also reduces the CYP3A4 enzyme inhibitory effect of the lead compound. In sum, it is considered to be an orally effective GnRH-A.

The pharmacokinetics studies showed Elagolix is rapidly absorbed after oral administration and its mean half-life (t1/2) is 2.4–6.3 h (Ng et al., 2017; Barra et al., 2019; Taylor et al., 2019). It is mainly processed through cytochrome P450 3A (CYP3A) mediated metabolism pathway in the liver. Also, 90% of its metabolites are discharged through feces (Ng et al., 2017). Additionally, the clinical studies found that the pharmacokinetic characteristics of Elagolix at 150 mg once a day and 200 mg twice a day seem not affected by factors including body weight, body mass index, race, or endometriosis status (Taylor et al., 2017; Surrey et al., 2018; Barra et al., 2019). There were two large-scale, double-blind, and stage III clinical trials (Elaris EM-I and Elaris EM-II) evaluated the efficacy of Elagolix in the treatment of endometriosis for 6 months (Taylor et al., 2017). The results showed that Elagolix exhibited good efficacy in endometriosis treatment. The major adverse reaction of taking Elagolix is the dose-dependent decrease in bone mineral density (Taylor et al., 2017).

Elagolix was approved by FDA in 2018 for the treatment of endometriosis-related pain. Infertility is a common complication of endometriosis, and the paradox is that most of the drugs used to treat endometriosis hinder fertility, so the development of drugs to treat endometriosis without obstructing fertility is a very promising direction. A clinical study of the use of Elagolix to pre-treat endometriosis patients with infertility before *In vitro* fertilization (IVF) treatment was initiated on 15 March 2022 (NCT04173169), with an expected primary completion date of 30 September 2024 (clinicaltrials.gov).

#### 3.3.2 SKI2496

**FIGURE 4:**
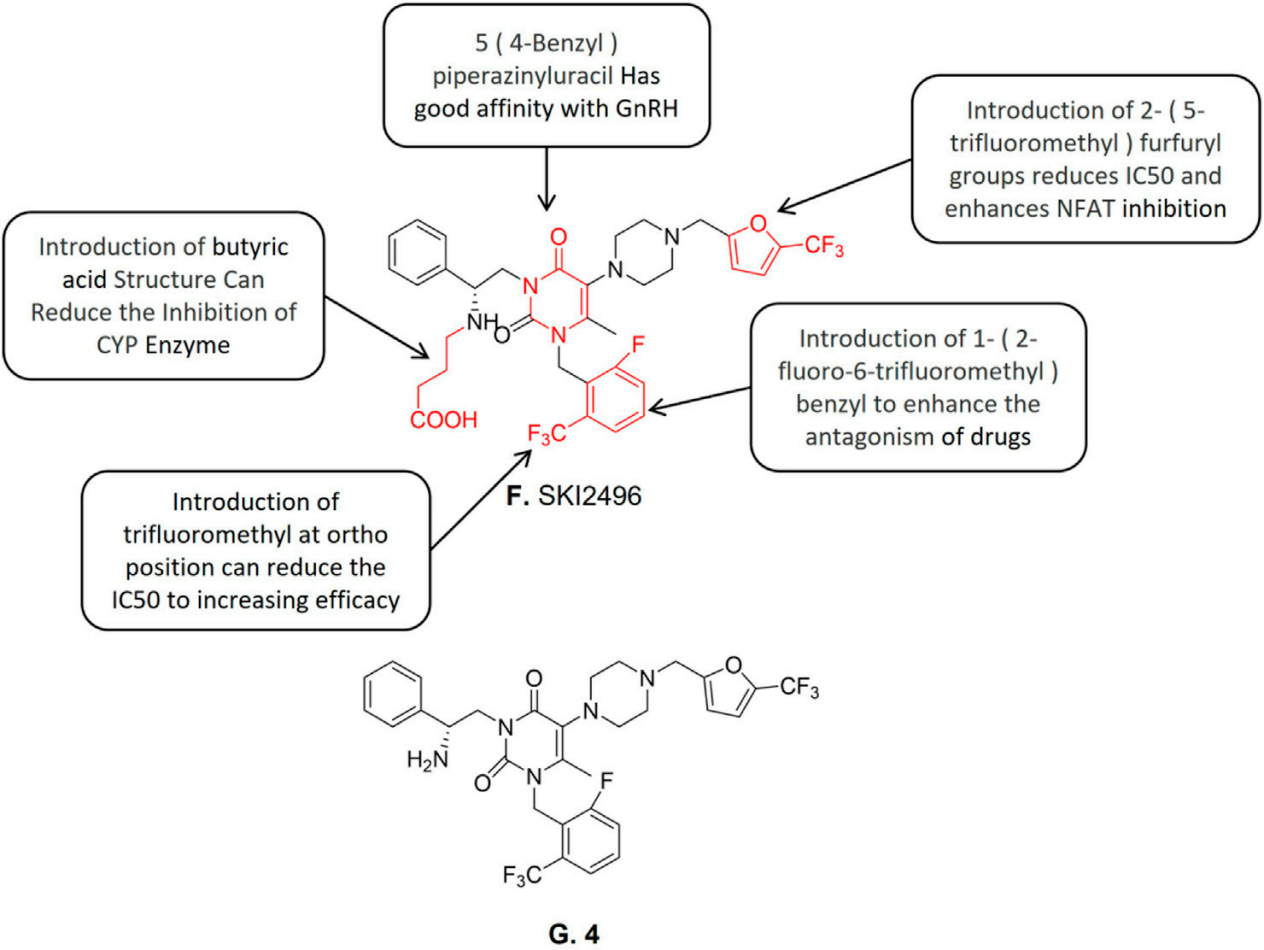
Structure-activity relationship of SKI2496 and structure of 4.

Compound SKI2496 (Figure 4F) is a non-peptide gonadotropin releasing antagonist. It is a promising candidate for oral hormone therapy of endometriosis treatment. Seon-Mi Kim et al. (Kim et al., 2016) developed a compound library of oral gonadotropin-releasing hormone (GnRH) receptor antagonists based on the uracil scaffold structure in the gonadotropin-releasing antagonist which plays a critical role. According to relevant reported structure-activity analyses (Guo et al., 2004; Betz et al., 2008; Gruen et al., 2014), urouracil scaffold is an essential active structure of gonadotropin releasing antagonists. A promising lead compound 4 (Figure 4G) was first identified with 4—(((5—trifluoromethyl) furan-2—yl)) piperazine group at the 5′ position, which exhibited the highest binding affinity (IC_50_ = 0.95 nM) and activation T nuclear factor (NFAT) inhibition activity (IC_50_ = 12.6 nM). The structure-activity relationship studies showed that the compounds containing 5- (4-benzyl) piperazine (IC_50_ = 12 nM) exhibited weak to zero affinity to hGnRH-R. The introduction of lipophilic electron-withdrawing groups at the 5- benzyl substituent position of uracil improved the compound’s affinity with GnRH-R therefore increasing its GnRH-R antagonist activity. Subsequent studies discovered that the introduction of 2-(5-trifluoromethyl) furfuryl reduces the IC_50_ value of NFAT inhibition and enhances the NFAT inhibition activity. However, the aforementioned modifications also made the new compound an effective CYP3A4 inhibitor. To solve this problem, an extended acid structure was added in three different regions of the uracil scaffold. Previous studies reported that the length of alkyl on the N-3 side chain affects the affects the activity of binding to the receptor and butyric acid seems to provide the best distance (Chen et al., 2008b). The introduction of butyric acid made the compounds significantly reduced the CYP3A4 inhibitory effect while retaining the hGnRH-R binding activity which is equivalent to the corresponding parent compounds. Based on the reported structure-activity relationships, the N-3 side chain of compound 14k was introduced into the pyrimidine scaffold to synthesize compound SKI2496. Further investigation on compound SKI2496s GnRH-induced downstream cell response was conducted. The results showed compound SKI2496 effectively blocked Ca^2+^ flux with an IC_50_ value of 0.76 nM. Besides, compound SKI2496 also showed ERK1/2 phosphorylation inhibitory activity with an IC_50_ value of 2.9 nM. The aforementioned results demonstrated that compound SKI2496 inhibits both GnRH-R activity and intracellular GnRH-mediated signaling pathways (Kim et al., 2016). Animal trials’ results further confirmed compound SKI2496 inhibits both the Ca^2+^ flux and ERK activation of GnRH-mediated signaling pathways. Besides, the animal trial results also demonstrated that compound SKI2496 exhibits a selective antagonistic effect on hGnRH-R than the ones in monkeys or rats. The pharmacokinetic evaluation of compound SKI2496 in rats and monkeys showed it has strong efficacy and long duration.

In view of these excellent characteristics of compound SKI2496 presents both *in vitro* and *in vivo*, it has entered the preclinical toxicological evaluations. We believe the investigations of compound SKI2496 for the endometriosis treatment also provides helpful insights for the future oral GnRH-A analogues development.

#### 3.3.3 BAY1214784

**FIGURE 5:**
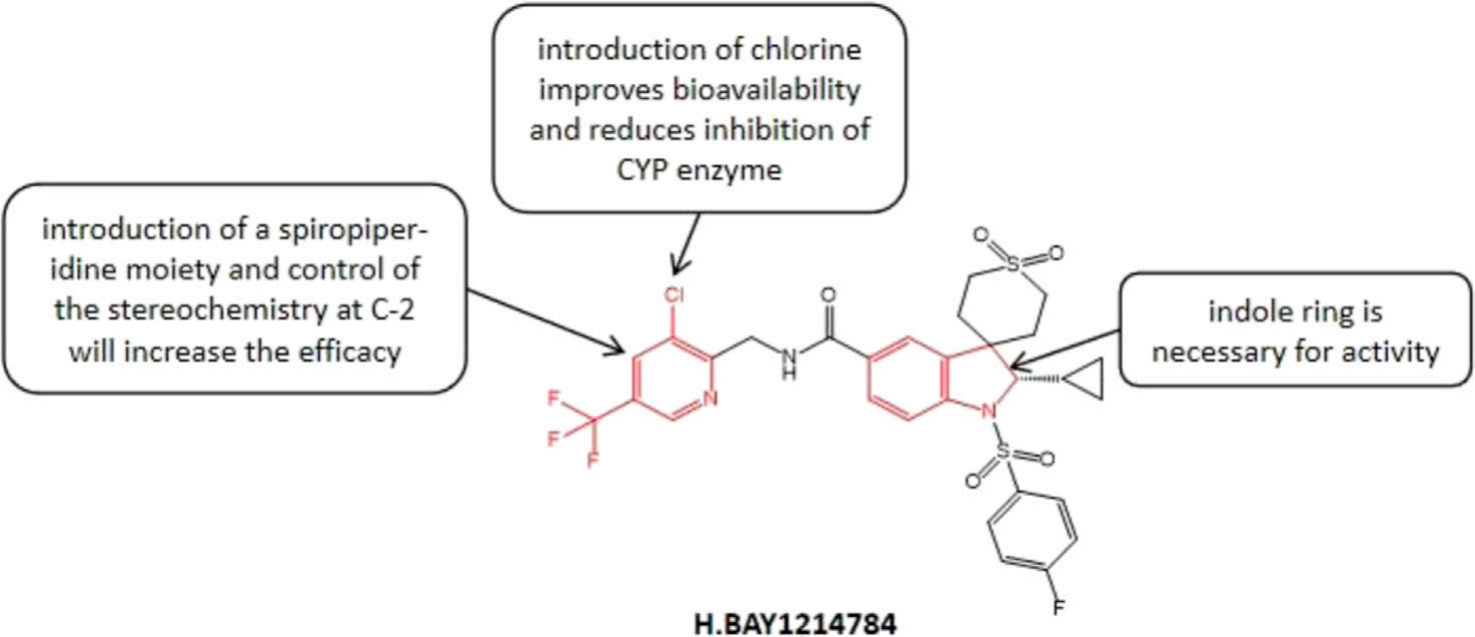
Structure-activity relationship of BAY1214784.

BAY1214784 (Figure 5) is an oral effective and highly selective hGnRH-A. Panknin et al. (Panknin et al., 2020) significantly improved the efficacy of GnRH-A in several species via changing the bismethylindoline core of the initial compound into a spiroindoline system, which is a necessary condition for the successful optimization of compounds under *in vivo* conditions. The structure-activity relationship studies showed that the introduction of spiroindoline structure significantly improved the therapeutic effect of lead compound. The introduction of chloropyridine groups into the amide side chain effectively alleviated the inhibition of the CYP enzyme and improved oral bioavailability. Related studies have shown that the single substitution of spirocyclic [piperidin-indoline] nucleus C-2 has a great influence on the GnRH-A antagonist effect. For example, with the introduction of a methyl at the C-2 position, compared with the C-2 unsubstituted derivatives, the determination of human luteinizing hormone releasing hormone increased five folds (Panknin et al., 2020). Usually, the hydrocarbons with lower space requirements are preferred at C-2, and the tolerance of polar substituents is poor (for example, the biological potency of hydroxyalkyl derivatives decreases by 58 folds compared with allyl derivatives). In general, the compounds introducing cyclopropyl into the spiro [piperidin-indoline] nucleus C-2 are considered have the best balance between potency and DMPK spectrum. The subsequent studies showed that the introduction of a chlorine atom at the 3- position of the pyridine ring increased the compound’s antagonistic effect against GnRH-R but reduced its lipophilicity. At present, a study evaluating the efficacy and safety of Linzagolix in subjects with moderate to severe endometriosis-related pain has been completed, however, the research results have not been disclosed yet.

### 3.4 Non-steroidal anti-inflammatory drugs (NSAIDs)

The NSAIDs’ pain-alleviating effects are reached through multiple mechanisms including inhibiting the prostaglandins synthesis, lymphocytes activity, and the differentiation of activated T lymphocytes. Besides, they also reduce the stimulation of afferent nerves and even directly affect the nociceptive receptors to prevent the production and release of pain-causing substances. NSAIDS exhibit excellent effects on primary dysmenorrhea and are widely used as first-line treatment for endometriosis-related pain (Dunselman et al., 2014). It is recommended to use NSAIDS or other analgesics to alleviate the endometriosis-related pain. However, patients must be aware that the long-term use of NSAIDs may lead to severe adverse reaction events including gastrointestinal ulcers, cardiovascular events, hypertension, and acute renal failure (Brown et al., 2017).

### 3.5 EP4 receptor antagonist

Inflammation is the key pathological process in endometriosis and often triggers pelvic pain. The prostaglandin E2 (PGE2) receptor (EP4-R) EP4 subtype is a particularly promising anti-inflammatory and anti-nociceptive target because PGE2 and this receptor subtype are highly expressed in endometrial lesions. Previous studies reported that the PGE2 synthesis and the EP4 subtype expression was significantly upregulated among the endometriosis-patients (Chishima et al., 2002; Chishima et al., 2007; Lousse et al., 2010; Santulli et al., 2014). EP4-R has been demonstrated to be responsible for mediating PGE2-induced proliferative and nociceptive effects, thus representing a valid target for specific intervention (Banu et al., 2009; Woodward et al., 2011). The antagonism of EP4 receptor exhibited various effects including blocking PGE2-induced endometrial cell proliferation (Lee et al., 2010; Woodward et al., 2011), modulating anti-inflammatory activities, (Woodward et al., 2011; Konya et al., 2013), and alleviating inflammatory pain. As the receptor is also expressed in sensory nociceptive nerve fibers and pain stimulation leads to an increase in expression in dorsal root ganglion (DRG) neurons (St-Jacques and Ma, 2014), antagonism of EP4-receptor can be expected to attenuate inflammatory pain and the subsequent induction of hyperalgesia and allodynia (Lin et al., 2006; Murase et al., 2008). Therefore, antagonistic EP4-R signal is a promising approach for treating endometriosis-related inflammatory pain.

#### 3.5.1 Bay1316957

**FIGURE 6:**
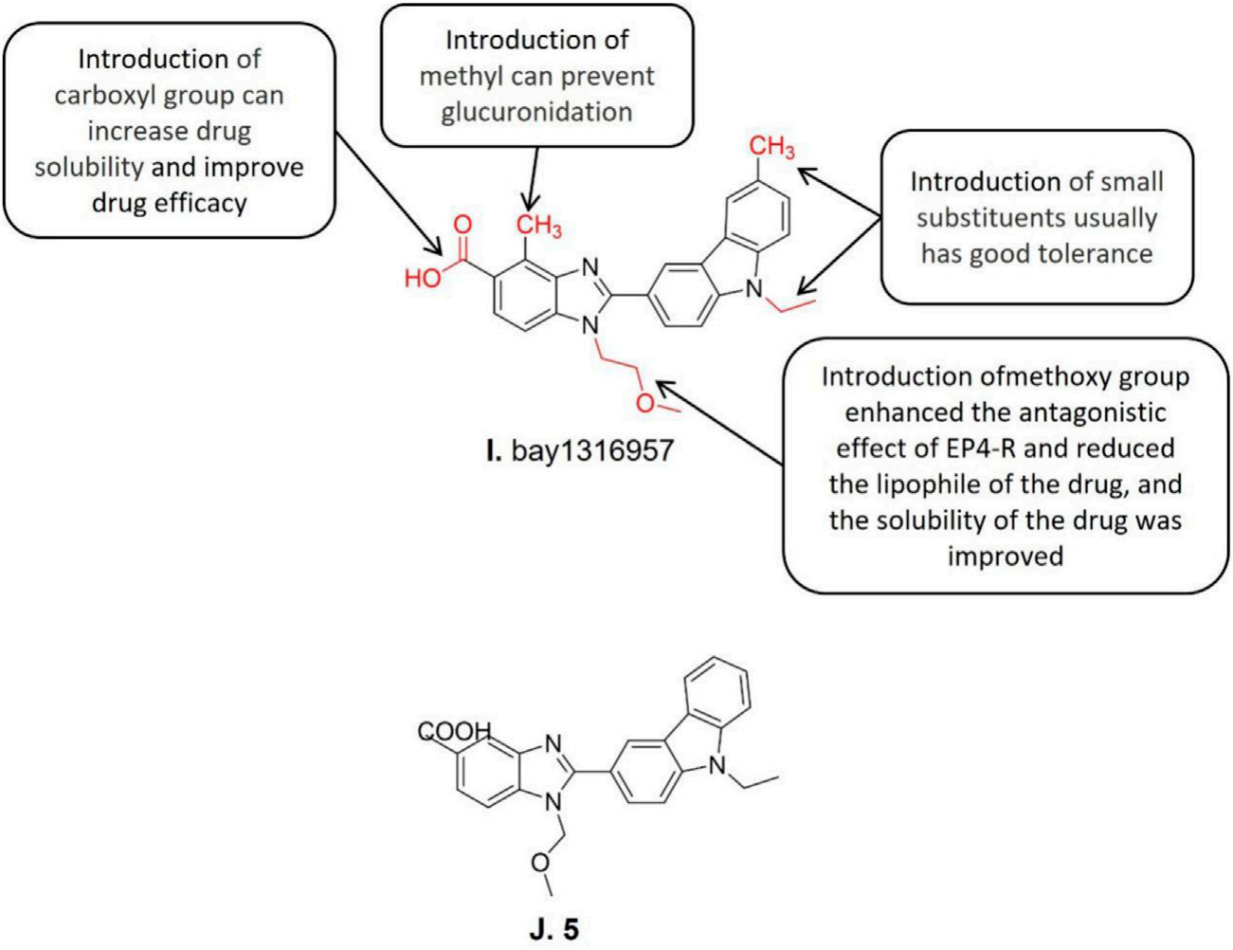
Structure-activity relationship and structures of bay1316957 and structure of 5.

Benzimidazole carboxylic acid derivatives represent a new class of efficient and highly selective EP4-R antagonists and bay1316957 (Figure 6I) is a typical lead compound of its kind (Baurle et al., 2019). The structure-activity relationship studies showed that the introduction of methoxy group into the nitrogen of benzimidazole in compound 5 (IC_50_ = 4.7 nM) (Figure 6J) enhanced the antagonistic effect against EP4–R, reduced the lipophilicity of the drug, and further improved the solubility. However, such modifications reduced its metabolic stability which the carboxylic acid part is easy to be glucuronated compared to its parental molecule. Subsequent studies further demonstrated that the introduction of carboxyl on the 5-position of the benzimidazole ring increases drug solubility and improves drug efficacy. Besides, the introduction of methyl at the adjacent position of carboxyl reduces the oxidation rate of carboxyl by glucuronidase. Based upon the aforementioned structure-activity relationships, the lead compound bay1316957 was synthesized after a series of structural modifications. The *in vitro* and *in vivo* studies on bay1316957 indicate it is a complete antagonist of human EP4-R with a series of excellent pharmacological features including low clearance rate, long half-life, and good oral bioavailability. What is more, bay1316957 exhibited excellent therapeutic activity in the *in vivo* abdominal pain model (Gruen et al., 2014)*.* It has been reported that bay1316957 exhibits superior pharmacokinetic profiles in rodents, which makes it suitable for the development of further endometriosis treatment (Baurle et al., 2019). The bay1316957 inhibits the EP4-R signaling pathway thereby triggers a series events as aforementioned which all contribute to the alleviation of the endometriosis inflammatory pain. To further optimize its application in treating endometriosis, more clinical practice like different combinations of bay1316957 with hormone drugs for treating endometriosis could be conducted. BAY 1316957 has excellent *in vitro* and *in vivo* efficacy and a superior pharmacokinetic profile, making it suitable for further development for the treatment of women with endometriosis.

### 3.6 SPRMs

Progesterone plays critical role in endometrial decidualization. SPRMs belong to the large progesterone receptor ligand family, and have mixed agonist–antagonist effects. There are a series of SPRMs which include Proellex, Mifepristone, Asoprisnil, and Vilaprisan, etc.

#### 3.6.1 Vilaprisan

**FIGURE 7:**
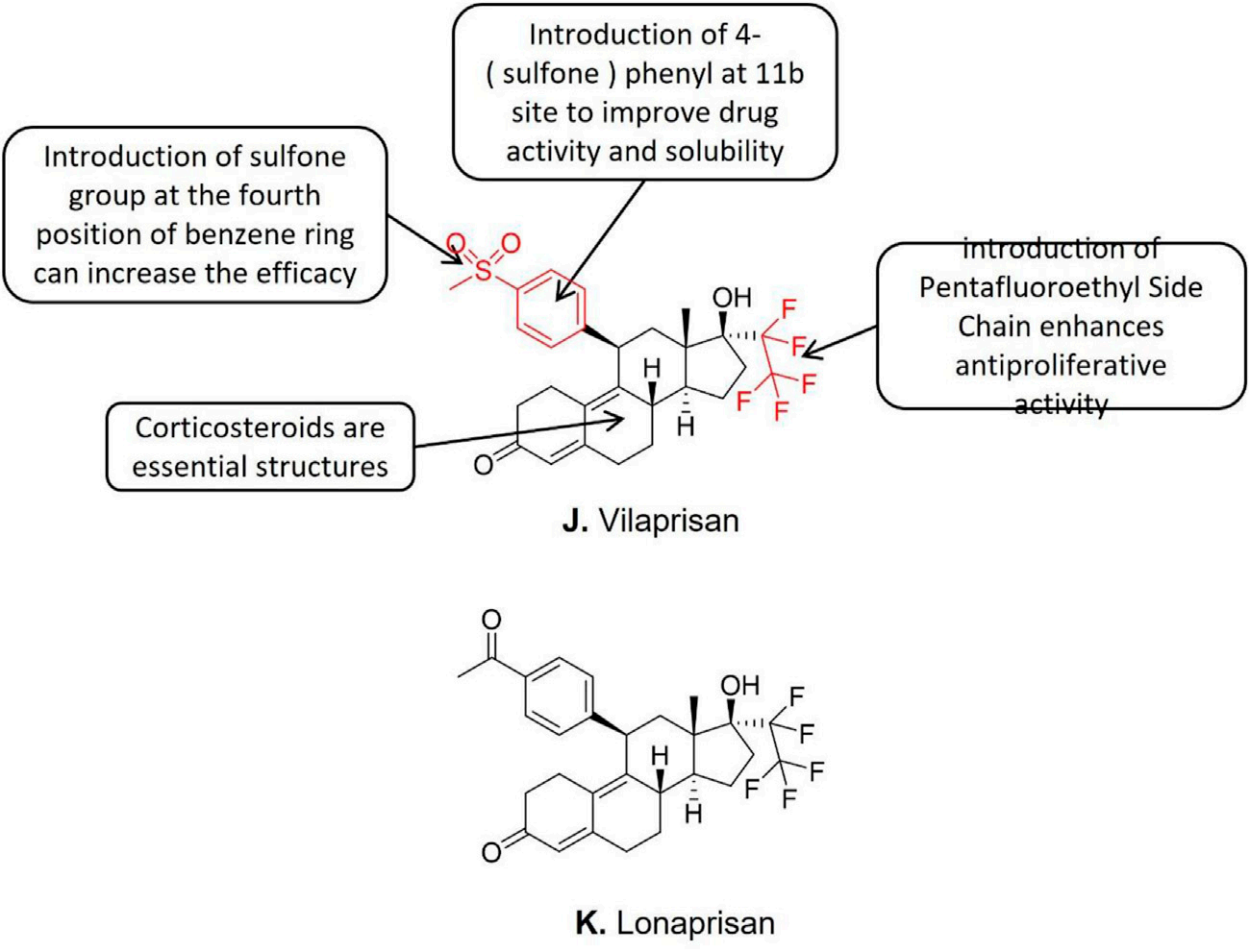
Conformational relationship of Vilaprisan and structure of Lonaprisan.

Bélanger et al. (Bélanger et al., 1981) discovered that the 11b phenyl is the critical structural element of all effective steroid PR modulators. Mifepristone is the earliest discovered and clinically applied SPRM, but severe adverse reactions like hepatotoxicity were reported. The structure-activity analysis disclosed that all the SPRM which exhibited hepatotoxicity contained the structure of 4—(dimethylamino) phenyls, such as mifepristone, ulistat acetate, and proellex (CDB4124). The dimethylamino structure is demethylated and further converted into aniline metabolites inside the hepatocytes which induces hepatotoxicity during the liver metabolism of SPRMs. The direct evidence of the SPRMs induced hepatotoxicity is the increased liver enzyme levels in blood tests after taking the relevant drugs (Möller et al., 2018). Further studies showed that SPRMs without dimethylaminophenyl (such as Lonaprisan) exhibited zero effects on liver enzyme activities (Möller et al., 2018). Lonaprisan generates three metabolites in the human bodthat enhance the activity and prolong the half-life of drugs (Möller et al., 2018). However, the long half-life of Lonaprisan (IC_50_ = 0.02 nM) elongates the circulation of its metabolites *in vivo*, which is considered to be a disadvantage for intermittent treatment. Therefore, Lonaprisan (Figure 7K) is not considered an ideal candidate for endometriosis treatment. Further modifications to the Lonaprisan molecule structure are needed. The structure-activity relationship studies showed that the introduction of carboxyl at 11b almost canceled the drug activity. Besides, the substitution with small amide groups at 11b improves its progesterone modulators’ activity and stability. What is more, the introduction of 4- (sulfone) phenyl at 11b avoids the hepatotoxicity of the original dimethylamino structure, and further improves the activity and solubility of the compound. Subsequent studies showed that the introduction of pentafluoroethyl at position 17a improves the selectivity of drugs to progesterone receptors.

Based upon the aforementioned reported structure-activity relationships a new SPRM named Vilaprisan (Figure 7J) was synthesized and Mçller et al. (Möller et al., 2018) reported that the IC_50_ value of Vilaprisan was 0.09 nM. In the transactivation test, although Vilaprisan exhibited lower modulatory activity of progesterone receptor compared to Lonaprisan, but Vilaprisan showed high stability in human and rat liver microsomes. Previous studies suggest Vilaprisan is an optimized SPRM for long-term and intermittent clinical application for premenopausal women (Möller et al., 2018). Vilaprisan entered clinical trial stage III for the endometriosis treatment, however, all the clinical studies of vilaprisan were suspended due to the discovery of drug toxicity signals in the long-term animal tests conducted in parallel with the Phase III clinical trial, even if the subsequent Phase II clinical data demonstrated the safety of the drug.

### 3.7 SERMs

Estrogen receptor (ER) has two subtypes, α and β. SERMs play a role similar to estrogen or anti-estrogen by binding to ER-α and/or ER-β in target cells. SERMs have tissue selectivity, and usually act as estrogen receptor agonists in bone and cardiovascular system, but work as antagonists of estrogen receptors in breast and endometrium (Bar et al., 2020). Endometriosis is an estrogen-dependent disease. SERM is designed to block the role of estrogen, as an endometriosis drug treatment (van Hoesel et al., 2021). Tamoxifen is the first generation SERM. However, occurrences of endometriosis were reported in postmenopausal patients who had been taking tamoxifen for treatment of breast cancer. And it had been suggested that long-term tamoxifen users are more likely to have endometrial hyperplasia, endometrial polyps, and/or endometrial cancer (Ford et al., 1988; Le Bouëdec et al., 1991).

SR-16234 is a selective estrogen receptor modulator (SERM) structurally different from approved SERM and has been reported to have estrogen receptor (ER) α antagonistic activity and a strong affinity with a weak partial agonistic activity to ERβ receptor (Harada et al., 2017). Its clinical application needs further study.

#### 3.7.1 Bazedoxifene

**FIGURE 8:**
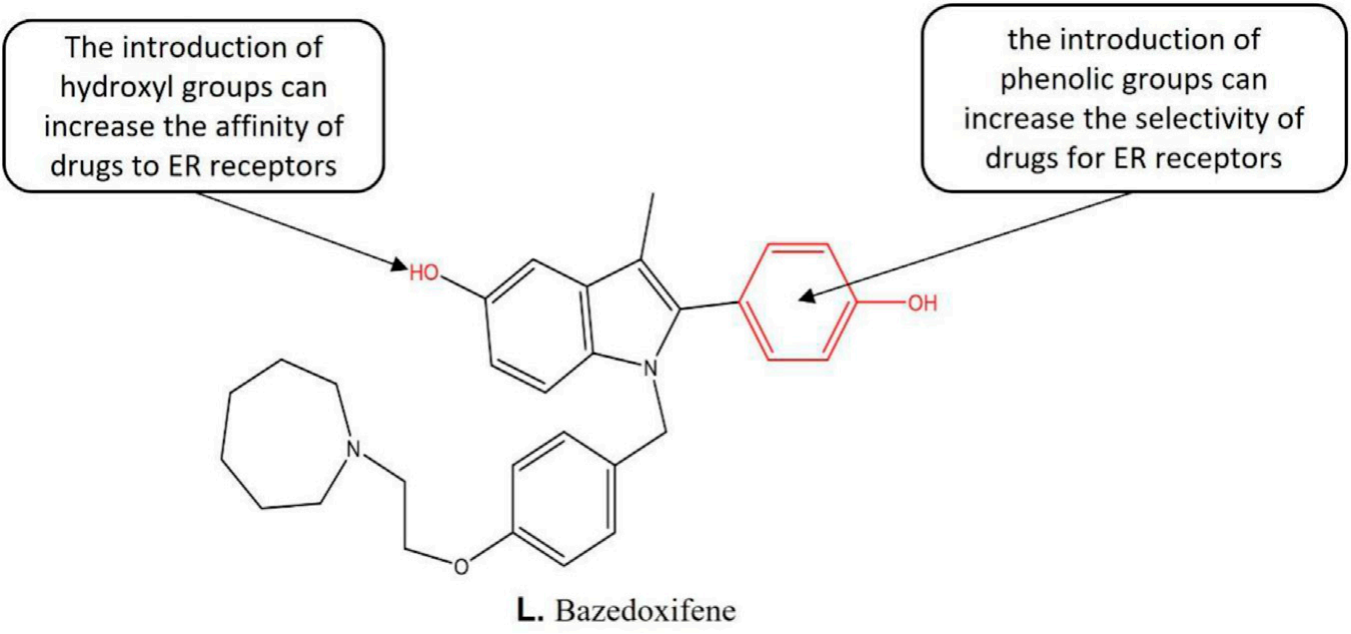
Structure-activity relationship of Bazedoxifene.

Bazedoxifene (BZA) (Figure 8) is a novel SERM drug for the treatment of osteoporosis in postmenopausal women with an increased risk of fracture. It effectively antagonizes estrogen-induced endometrial proliferation without countering estrogenic effects in bone or the central nervous system. These properties make it an attractive drug candidate for the treatment of endometriosis. Structure-activity relationship studies indicate that the introduction of phenolic groups increases the selectivity of drugs for ER receptors (Komm et al., 2014). The introduction of hydroxyl groups increases the affinity of drugs to ER receptors. In addition, both *in vitro* and animal studies have shown that BZA causes regression of endometriotic lesions (Kulak et al., 2011; Sakr et al., 2014), inhibits estrogen-mediated cell proliferation (Kulak et al., 2011) and reduces stem cell recruitment within endometriotic lesions (Sakr et al., 2014). However, the effectiveness of BZA for the treatment of pain associated with endometriosis remains to be investigated. The relevant studies have shown that BZA is more likely to demonstrate clinical efficacy than raloxifene. In sum, BZA has superior clinical efficacy and presents a novel agent for the endometriosis treatment.

### 3.8 AI

AI specifically inactivates aromatase by blocking the aromatization reactions, therefore inhibiting estrogen production, and finally reducing the estrogen level in the blood. Studies have confirmed that aromatase plays the ultimate rate-limiting catalytic role in estrogen biosynthesis and estrogens are associated with endometriosis development. The application of AIs for endometriosis treatment is based on the evidence of aromatase activity in ectopic endometrial lesions and the relationship between extrauterine endometrial tissue and serum estrogen levels. Aromatase activity is absent in normal human endometrium but elevated in endometriosis-tissues (Burney and Giudice, 2012). Studies have shown that extra-uterine endometrial tissue is a source of estrogen. Besides, estrogen stimulates the synthesis of PGE2, which is a potent inducer of ectopic endometrial aromatase activity. This is a potential mechanism for triggering a vicious cycle leading to new growth of ectopic endometrial tissue (Carvalho et al., 2011). Applying Ais for treating endometriosis can theoretically terminate this vicious cycle. There are three generations of commonly used Ais. Aminolumet is the first generation of Ais. The second-generation inhibitors include fadrozole and formestane. Anastrozole, letrozole, and exemestane are the third-generation inhibitors.

#### 3.8.1 Letrozole

**FIGURE 9:**
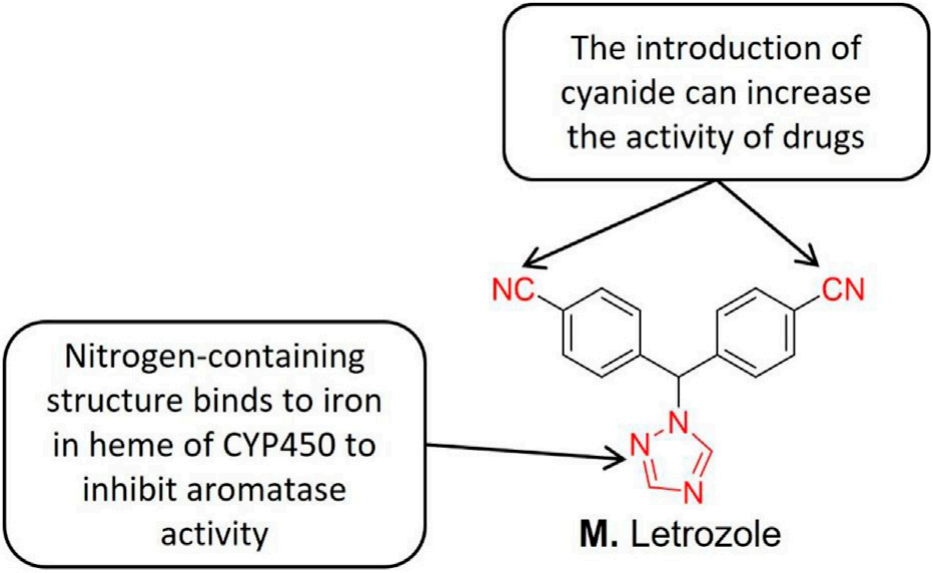
Structure-activity relationship of Letrozole.

Letrozole (Figure 9) is a triazole derivative of AI with high selectivity and reversibility. The triazole compound letrozole is superior to other farrezole derivatives in inhibiting AR *in vivo*. Structure-activity relationship studies indicate that the nitrogen-containing heterocyclic (such as triazole and imidazole) binds to the iron in the heme part of CYP450, thereby inhibiting AR activity (Bhatnagar, 2007). The nitrogen-containing heterocyclic group also inhibits *in situ* AR activity, reduces the endogenous estrogen secretion, reduces its content, and thus effectively promotes ovulation. Zhou et al. [126] reported that letrozole exhibits a strong ovulation induction effect thereby improving the fertility of infertile endometriosis patients. Zhou and his group further reported that laparoscopic surgery combined with ovulation induction treatment improved the fertility of patients with mild endometriosis. At present, letrozole has been applied to treat endometriosis patients (Lall Seal et al., 2011).

### 3.9 Natural products

#### 3.9.1 Resveratrol

Resveratrol is a polyphenol with anti-inflammatory effects (Lin et al., 1998). It exerts anti-inflammatory effects by inhibiting the release of cytokines (TNF-α, IL-6, IL-8, VEGF, and MCP-1) and the production of reactive oxygen species (ROS) in monocytes, macrophages, and lymphocytes. Bruner-Tran and Osteen (Herington et al., 2011) investigated Resveratrol’s endometriosis treatment effects in mice models. In this study, slow-release estrogen capsules were administered to ovariectomized nude mice 24 h before intraperitoneal injection of normally proliferating human endometrial tissue. A 6 mg/mouse dose of resveratrol was started 24 h after tissue injection and continued for 20 days. The results showed that the lesions of rats treated with resveratrol were less. The resveratrol did not appear to affect the apoptotic activity in the uteri of the cured mice. Although the resveratrol doses used in the study were significantly higher than the normal intake of resveratrol-containing nutrients, the above findings may suggest a possible role for resveratrol supplementation in the endometriosis treatment (Taylor et al., 2011).

### 3.10 Immunotherapy

Immune disorders play an important role in the implantation, adhesion, and proliferation of the endometriosis lesions. The inflammatory environment allows the peritoneal surface to provide a local microenvironment for retrograde endometrial, progenitor, or stem cell adhesion (Zondervan et al., 2018). Immune cells such as macrophages, thymus dependent lymphocytes (TDL) and their inflammatory mediators are involved in the pathogenesis of endometriosis (Chen et al., 2019).

#### 3.10.1 Interleukin (IL)

Interleukin (IL) plays critical roles in various immune response activities including transmitting information, activating, and regulating immune cells. In addition, it closely participates in the inflammatory response. Toru Kato et al. (Kato et al., 2019) used the endometriosis mouse model studied the role of the IL-1/IL-33 signaling pathway in endometriosis development. Mice injected intraperitoneally with anti-IL-1 receptor 1 (IL-1R1) or IL-33 antibodies showed limited endometrial lesions. Oral administration of IL-1 receptor 1 (IL-1R1) -associated kinase 4 (IRAK4) inhibitors also inhibited the formation of endometriosis lesions, and even prevented the expansion of lesions even after cystic lesions occurrance. These treatments decreased Ki-67 expression, and thus reduced cell proliferation. The study reveals that IL-1/IL-1R1, IL-33/IL-33R are involved in the pathogenesis of endometriosis, and may provide novel therapeutic targets for endometriosis (Kato et al., 2019).

Interleukin (IL)-33 is elevated in the plasma, peritoneal fluid, and endometriotic lesions of endometriosis patients. Miller et al. (Miller et al., 2021) reported that IL-33 contributes to the expansion of the novel group 2 innate lymphoid cells (ILC2s) which modulate the endometriosis lesion microenvironment. A Phase II clinical trial on the safety and efficacy of monoclonal antibody targeting IL-33 (MT-2990) in patients with endometriosis has been completed (Miller et al., 2021). However, its results have not been announced yet.

### 3.11 Dopamine (DA) receptor antagonist

The pathological hallmark of endometriosis is angiogenesis. In experimental models of endometriosis, the DAs (bromocriptine, cabergoline, and quinagolide) downregulate the pro-angiogenic pathway and upregulate the anti-angiogenic pathway in inflammatory cells, endothelial cells, and endometrial cells which result in blocking cell proliferation and reducing lesion size (Pellicer et al., 2021). Maria Yarmolinskaya et al. (Yarmolinskaya et al., 2020) demonstrated that the intravaginal administration showed higher bioavailability than oral administration throughout the menstrual cycle of up to 35 days. Therefore, transvaginal administration of DAs may become an option for the endometriosis treatment.

#### 3.11.1 Quinagolide

**FIGURE 10:**
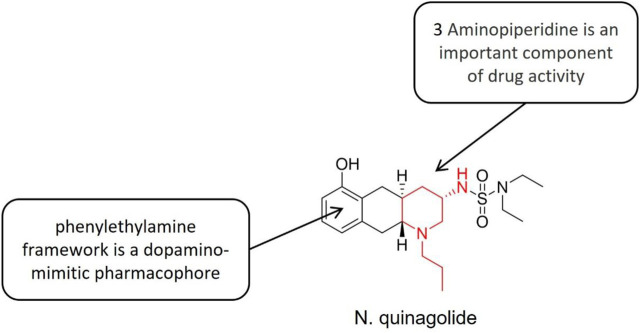
Structure-activity relationship of quinagolide.

Quinagolide (Figure 10) is a powerful dopamine receptor (D1, D2) agonist, which inhibits the synthesis and release of prolactin (PRL) through receptor function at the level of prolactin (PRL) cells in the hypothalamus-pituitary axis (HP axis). It presents strong and persistent effects, good tolerance and mild side effects. The D1 category promotes increased adenylate cyclase activity and the D2 category not changing or decrease it. The structure-activity relationship study showed that the phenylethylamine structure was a dopamine quasi-pharmacological group, and the nitrogen-containing side chain located at the C-3 standard increased the selectivity to D2 receptor (Chavan et al., 2021). In the experimental model of endometriosis, quinagolide downregulated the proangiogenic pathway and upregulated the antiangiogenic pathway, to reduce angiogenesis. Besides, it also blocked cell proliferation and reduced lesion size (Pellicer et al., 2021). The vaginal ring of quinagolide was well tolerated and did not change the level of reproductive hormones, menstrual cycle, or endometrial histology.

A clinical study about Quinagolide was initiated on 21 November 2018, and the results were posted on 7 September 2022 (NCT03749109). The main data used for drug efficacy evaluation were the sizes of Endometrioma, deep Infiltrating endometriosis, and adenomyosis lesions summed by type on magnetic resonance Images at menstrual Cycle 4. However, no statistical difference between the study group and the placebo group (clinicaltrials.gov).

## 4 Prospective

Current treatments for endometriosis include surgical removal of the lesion and pharmacological suppression of ovarian hormone, surgery combined with pharmacological treatment, and assisted reproductive techniques. More than half of the women who took surgery will need additional surgical procedures 5 years later (Saraswat et al., 2018). The first-line treatment for endometriosis is a combination of oral contraceptives and progestins (Vercellini et al., 2014) which provides effective pain management and is easily tolerated by patients. However, the endometriosis induced pain is partially relieved or unrelieved or recurs after stopping treatment. What is more, these treatments prevent the patient from becoming pregnant. In the second-line drugs for endometriosis treatment, GnRH-a has been widely used in the last decade. However, GnRH-a causes varying degrees of adverse effects to including reduced bone density and hot flashes because of low estrogen. Researchers have investigated the inhibition of other pathways that cause endometriosis to identify the potential new drugs for endometriosis treatment. Such efforts have led to the development of several potential candidates includingng GnRH-As, AIs.

The GnRH-A is an important research direction in endometriosis treatment. The treatment course is short and the curative effect is good, so it has great application prospects for endometriosis. AIs reduce estrogen levels, promote ovulation, and improve the fertility of patients with endometriosis. It is hoped that the new drugs developed in the future better alleviate the endometriosis symptoms, reverse infertility in the patients, with less adverse reactions, and avoid recurrence, thus significantly improving the endometriosis patient’s life quality, to achieve individualized administration and more effective treatment of endometriosis.
