# Corrigendum: Research advances in drug therapy of endometriosis

**DOI:** 10.3389/fphar.2023.1254518

**Published:** 2023-07-14

**Authors:** Jianyou Shi, Xin Tan, Guimei Feng, Yuan Zhuo, Zhongliang Jiang, Srikanth Banda, Lin Wang, Wei Zheng, Lu Chen, Dongke Yu, Chun Guo

**Affiliations:** ^1^ Department of Pharmacy, Sichuan Academy of Medical Sciences and Sichuan Provincial People’s Hospital, School of Medicine, University of Electronic Science and Technology of China, Chengdu, China; ^2^ Personalized Drug Therapy Key Laboratory of Sichuan Province, School of Medicine, University of Electronic Science and Technology of China, Chengdu, China; ^3^ Center for Reproductive Medicine, Department of Obstetrics and Gynecology, Sichuan Provincial People's Hospital, University of Electronic Science and Technology of China, Chengdu, China; ^4^ Chinese Academy of Sciences Sichuan Translational Medicine Research Hospital, Chengdu, China; ^5^ Pharmacy College, Chengdu University of Traditional Chinese Medicine, Chengdu, China; ^6^ Miller School of Medicine, University of Miami, Miami, FL, United States; ^7^ Department of Chemistry and Biochemisty, Florida International University, Miami, FL, United States; ^8^ College of Food and Bioengineering, Xihua University, Chengdu, China

**Keywords:** endometriosis, pathogenesis, medical treatment, gonadotropin-releasing antagonist, aromatase inhibitor

In the published article, there was an error in **affiliation** 3. Instead of “Chinese Academy of Sciences Sichuan Translational Medicine Research Hospital, Chengdu 610072, China”, it should be “Center for Reproductive Medicine, Department of Obstetrics and Gynecology, Sichuan Provincial People’s Hospital, University of Electronic Science and Technology of China, Chengdu, China”. There was also an error in **affiliation** 4. Instead of “Center for Reproductive Medicine, Department of Obstetrics and Gynecology, Sichuan Provincial People’s Hospital, University of Electronic Science and Technology of China, Chengdu, China”, it should be “Chinese Academy of Sciences Sichuan Translational Medicine Research Hospital, Chengdu, China.

The authors apologize for this error and state that this does not change the scientific conclusions of the article in any way. The original article has been updated.

