# Corrigendum: Research advances in drug therapy of endometriosis

**DOI:** 10.3389/fphar.2023.1274946

**Published:** 2023-08-23

**Authors:** Jianyou Shi, Xin Tan, Guimei Feng, Zhou Yuan, Zhongliang Jiang, Srikanth Banda, Lin Wang, Wei Zheng, Lu Chen, Dongke Yu, Chun Guo

**Affiliations:** ^1^ Department of Pharmacy, Sichuan Academy of Medical Sciences and Sichuan Provincial People’s Hospital, School of Medicine, University of Electronic Science and Technology of China, Chengdu, China; ^2^ Personalized Drug Therapy Key Laboratory of Sichuan Province, School of Medicine, University of Electronic Science and Technology of China, Chengdu, China; ^3^ Center for Reproductive Medicine, Department of Obstetrics and Gynecology, Sichuan Provincial People’s Hospital, University of Electronic Science and Technology of China, Chengdu, China; ^4^ Chinese Academy of Sciences Sichuan Traditional Medicine Research Hospital, Chengdu, China; ^5^ Pharmacy College, Chengdu University of Translational Chinese Medicine, Chengdu, China; ^6^ Miller School of Medicine, University of Miami, Miami, FL, United States; ^7^ Department of Chemistry and Biochemistry, Florida International University, Miami, FL, United States; ^8^ College of Food and Bioengineering, Xihun University, Chengdu, China

**Keywords:** endometriosis, pathogenesis, medical treatment, gonadotropin-releasing antagonist, aromatase inhibitor

In the published article, there was an error in Figure 5 as published. In Figure 5, the structure and name of the compound are incorrect. The corrected Figure 5 appears below.

**FIGURE 5:**
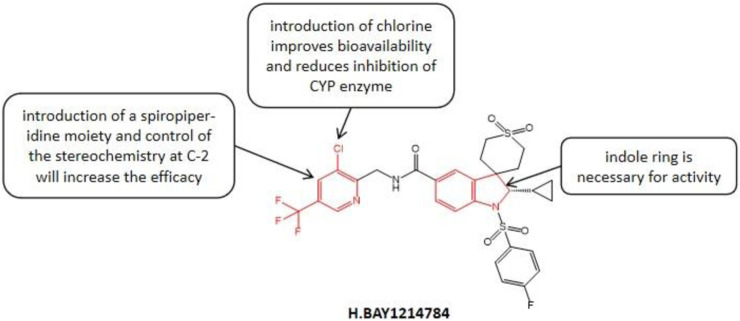
Structure-activity relationship of BAY1214784.

In the published article, the reference for **Donnez et al., 1994** was incorrectly written as “Donnez, J., Nisolle, M., Clerckx, F., Casanas-Roux, F., Saussoy, P., and Gillerot, S. (1994). Advanced endoscopic techniques used in dysfunctional bleeding, fibroids and endometriosis, and the role of gonadotrophin-releasing hormone agonist treatment. Br J Obstet Gynaecol 101 Suppl 10, 2-9. doi: 10.1111/j.1471-0528.1994.tb13677.x.” It should be deleted.

In the published article, the reference for **Carr et al., 2014** was incorrectly written as “Carr, B., Dmowski, W.P., O'Brien, C., Jiang, P., Burke, J., Jimenez, R., et al. (2014). Elagolix, an oral GnRH antagonist, versus subcutaneous depot medroxyprogesterone acetate for the treatment of endometriosis: effects on bone mineral density. Reprod Sci 21(11), 1341-1351. doi: 10.1177/1933719114549848.” It should be deleted.

In the published article, the reference for **Donnez et al., 2020** was incorrectly written as “Donnez, J., Taylor, H.S., Taylor, R.N., Akin, M.D., Tatarchuk, T.F., Wilk, K., et al. (2020). Treatment of endometriosis-associated pain with linzagolix, an oral gonadotropin-releasing hormone-antagonist: a randomized clinical trial. Fertil Steril 114(1), 44-55. doi: 10.1016/j.fertnstert.2020.02.114.” It should be deleted.

A correction has been made to the **Heading** of **3.3.3**. This heading previously stated:

“3.3.3 Linzagolix”

The corrected sentence appears below:

“3.3.3 BAY1214784”

A correction has been made to **3.3.3**, paragraph 1. This sentence previously stated:

“Linzagolix (Figure 5) is an oral effective and highly selective hGnRH-A.”

The corrected sentence appears below:

“BAY1214784 (Figure 5) is an oral effective and highly selective hGnRH-A.”

A correction has been made to **3.3.3**, paragraph 2. This sentence previously stated:

The pharmacokinetics studies further demonstrated BAY1214784 exhibits excellent features *in vivo* including an appropriate half-life of 15–18 h, high oral bioavailability, small amount of distribution, zero food effect, and no interaction with transporters or CYP3A4 enzymes (**Donnez et al., 1994**; **Carr et al., 2014**). Donnez et al. (**Donnez et al., 2020**) investigated the effect of BAY1214784 on treating endometriosis-related pain (EAP). The results showed that compared with placebo, BAY1214784 significantly alleviated the endometriosis-associated pain in the patients.

The sentences below need to be deleted. After the above sentences are deleted, the last sentence of paragraph 2 is merged into the first paragraph.

The authors apologize for these errors and state that this does not change the scientific conclusions of the article in any way. The original article has been updated.

